# Toll‐like receptor involvement in adolescent scoliotic facet joint degeneration

**DOI:** 10.1111/jcmm.15733

**Published:** 2020-08-27

**Authors:** Daniel G. Bisson, Kai Sheng, Semsi Kocabas, Emerson Krock, Alisson Teles, Neil Saran, Jean A. Ouellet, Lisbet Haglund

**Affiliations:** ^1^ Shriners Hospital for Children Montreal QC Canada; ^2^ Orthopaedic Research Laboratory Department of Orthopedic Surgery McGill University Montreal QC Canada

**Keywords:** adolescent idiopathic scoliosis, cartilage, cytokines, extracellular matrix, facet joint, metalloproteases, osteoarthritis

## Abstract

Facet joint osteoarthritis is prevalent in young patients with adolescent idiopathic scoliosis (AIS) and might contribute to back pain. Toll‐like receptors (TLR) have been linked to cartilaginous tissue degeneration but their involvement in facet joint osteoarthritis in AIS patients is still unknown. We compared baseline gene expression levels of TLRs ‐1, ‐2, ‐4, and ‐6 in scoliotic and non‐scoliotic chondrocytes and found higher expression levels in scoliotic chondrocytes with significantly higher TLR2 levels. Furthermore, TLR expression correlated strongly and significantly with inflammatory and catabolic markers in scoliotic but not in non‐scoliotic chondrocytes. TLR activation with a synthetic TLR2/6 agonist resulted in a robust induction and release of pro‐inflammatory and catabolic factors which exacerbated proteoglycan loss in scoliotic but not in non‐scoliotic cartilage. We also detected a higher abundance of alarmins including S100A8/9 and biglycan in scoliotic cartilage. Finally, the small‐molecule antagonists Sparstolonin B and o‐Vanillin reduced catabolism following induction with naturally occurring alarmins and the synthetic TLR2/6 agonist. The high baseline expression, robust responsiveness and strong and significant correlation with proteases and pro‐inflammatory cytokines suggest that TLRs are key regulators of facet joint degeneration in AIS. Blocking their activity could therefore potentially modify disease progression.

## 1 INTRODUCTION

Zygapophyseal also known as facet joints are synovial joints located on each side of the posterior spinal column of each motion segment. They play a primary role in spinal stabilization and load bearing. Studies show facet joints can carry up to 25% of the axial compression and 40%‐65% of torsional and shearing forces in a healthy spine.^1^, ^2^, ^3^ Balanced load dispersion between the intervertebral discs (IVD) and both facet joints is important to prevent degeneration of these cartilaginous tissues. Many studies have confirmed the detrimental effects of non‐physiological loading on both facet joints and IVDs, as well as segmental degeneration following injurious loading. One such example is the presence of IVD and facet joint degeneration in adolescent idiopathic scoliosis (AIS).^4^, ^5^, ^6^ AIS is the most prevalent orthopaedic condition (up to 4%) in young individuals aged from 10 to 16 worldwide that involves a progressive curvature of the spine.^7^ The imbalanced load results in wedging that causes disc degeneration, which is thought to contribute to coronal deformation.^8^ Similarly, we have recently reported facet joint osteoarthritis (OA) in AIS patients, although, how the altered biomechanics affect the facet joints is still unclear. Histologically, we found that scoliotic and non‐scoliotic facet joint OA share many characteristics such as fibrillation of the cartilage surface, erosion, fissuring, osteophyte formation, increased cell density and elevated expression of pro‐catabolic and pro‐inflammatory factors which suggests that they are closely related.^4^, ^9^, ^10^

The underlying pathology of facet joint OA is a catabolic and inflammatory environment that leads to degradation, loss of function and potentially pain. Pro‐inflammatory cytokines are secreted by chondrocytes and synovial fibroblasts and cause catabolism by triggering the production of proteases degrading the extracellular matrix (ECM).^11^, ^12^, ^13^ Interestingly, a growing body of literature links toll‐like receptors (TLR) to cartilaginous tissue degeneration.^14^, ^15^, ^16^ TLRs are a family of pattern recognition receptors with 10 members in human cells. They are activated by a variety of molecules derived from pathogens, endogenous danger related proteins released by stressed cells or fragmented ECM components. These endogenous proteins such as HSP60, HSP70, S100A8/9, biglycan, HMGB1, fibronectin and aggrecan‐fragment belong to a group called *alarmins* and have been measured in higher abundance in degenerating cartilage.^17^, ^18^, ^19^, ^20^, ^21^, ^22^, ^23^ TLR ligands selectively bind specific TLR hetero‐ or homo‐dimers and generate an inflammatory downstream response.^24^ Although TLR1‐9 protein expression was found to correlate with degenerative changes in adult OA chondrocytes, most of the alarmins bind to either TLR2 which dimerizes with TLR1 and 6, or to TLR4‐heterodimers.^25^ TLR homo‐ or hetero ‐dimerization generally results in recruitment and activation of MyD88 through the Toll/IL‐1R homology domain. This leads to the activation of the transcription factor NF‐kB and downstream expression of pro‐inflammatory, catabolic and pro‐nociceptive factors.^16^, ^24^, ^26^, ^27^, ^28^ There has been a recent interest in using small‐molecule inhibitors to block TLR activation. Two such molecules are o‐Vanillin and Sparstolonin B which interfere with MyD88 recruitment preventing downstream signalling.^29^, ^30^, ^31^

This study is focused on evaluating a potential involvement of the TLRs in AIS facet joint OA and evaluating the potential of inhibiting TLRs using small‐molecule inhibitors as potential disease‐modifying therapeutics.

## 2 MATERIALS AND METHODS

### 2.1 Sample collection and processing

Facet joints were resected with consent from AIS patients undergoing corrective surgery. Non‐scoliotic thoracolumbar facet joints were from organ donors without known spinal pathology and were obtained with consent through a collaboration with Transplant Quebec. A total of 18 scoliotic patients and 12 non‐scoliotic organ donors were included in this study, with a mean age of 15.0 ± 1.12 and 41.5 ± 9.66, respectively. From each donor, one left and right upper facet joint from the same spinal level were collected and processed (Table 1). The facet joint cartilage was removed from the subchondral bone and was either digested with Collagenase Type II (Gibco) to release chondrocytes^32^ or cultured as explants^33^ in complete chondrocyte media (Dulbecco's DMEM with 4.5 g/L glucose (Sigma Aldrich), 10% FBS, 25 µg/mL gentamycin (Life Technologies), 2 mmol/L Glutamax (Life Technologies)). Collagenase digestion generated on average 1 × 10^6^ chondrocytes per 100 mg of tissue. The study was approved by the McGill University institutional review board in Montreal, Canada (IRB # Tissue Biobank 2019‐4896, Extracellular Matrix 2020‐564 and A08‐M22‐17B).

**Table 1:** Tissue donor information for both scoliotic and non‐scoliotic groups

Group	Age	Sex	Major curve cobb angle (°)	Cause of death
Scoliotic	14	Female	68	
Scoliotic	13	Female	67	
Scoliotic	17	Female	45	
Scoliotic	14	Female	56	
Scoliotic	15	Female	50	
Scoliotic	17	Female	55	
Scoliotic	19	Male	50	
Scoliotic	13	Male	78	
Scoliotic	18	Male	62	
Scoliotic	14	Female	50	
Scoliotic	17	Male	50	
Scoliotic	13	Female	70	
Scoliotic	13	Female	64	
Scoliotic	14	Female	50	
Scoliotic	15	Female	60	
Scoliotic	13	Female	55	
Scoliotic	19	Male	55	
Scoliotic	12	Female	48	
Non‐scoliotic	60	Female		Stroke
Non‐scoliotic	34	Male		Stroke
Non‐scoliotic	49	Female		Haemorrhage
Non‐scoliotic	53	Male		Haemorrhage
Non‐scoliotic	17	Male		Anoxia
Non‐scoliotic	22	Male		Anoxia
Non‐scoliotic	53	Female		Anoxia
Non‐scoliotic	28	Male		Anoxia
Non‐scoliotic	27	Male		Trauma
Non‐scoliotic	42	Male		Stroke
Non‐scoliotic	57	Male		Haemorrhage
Non‐scoliotic	56	Male		Anoxia

### 2.2 Gene expression analysis

Freshly isolated scoliotic (n = 18 from 9 donors) and non‐scoliotic (n = 12 from 6 donors) chondrocytes were lysed with TRIzol (Life technologies). RNA was extracted according to the manufacturer's instructions and re‐suspended in DEPC water (Life technologies). 1 µg of RNA was reverse transcribed with the Reverse‐transcriptase kit (Life technologies). RT‐qPCR was performed on a QuantStudio 7 (Life Technologies) using PowerUp Sybr Green master mix (Life Technologies) and primers listed in Table 2. The results were analysed by the comparative CT method using ß‐actin as the reference gene. When compared to untreated or alarmin‐treated controls, the fold change was calculated using the 2^−ddCT^ method and normalized to ß‐actin.

**Table 2:** Human oligonucleotides used in gene expression analysis

Gene of interest	Forward primer	Reverse primer
TLR1	cagtgtctggtacacgcatggt	tttcaaaaaccgtgtctgttaagaga
TLR2	ggccagcaaattacctgtgtg	Aggcggacatcctgaacct
TLR4	cagagtttcctgcaatggatca	gcttatctgaaggtgttgcacat
TLR6	gaagaagaacaaccctttaggatagc	aggcaaacaaaatggaagctt
MMP3	aatggcattcagtccctctatg	Gacaggttccgtgggtac
MMP13	gatgacgatgtacaagggatcc	Agggtcacatttgtctggc
IL‐1ß	aagcttggtgatgtctggtc	acaaaggacatggagaacacc
IL‐6	tgaaccttccaaagatggctg	caaactccaaaagaccagtgatg
IL‐8	tcctgatttctgcagctctg	gtctttatgcactgacatctaagttc
Actin	gtcttcccctccatcgtgg	Aatccttctgacccatgcc

### 2.3 Ex vivo* culture and TLR activation*

Cartilage explants of scoliotic (n = 15 donors) and non‐scoliotic (n = 7 donors) facet joints were generated with a 8 mm biopsy punch. The biopsy was divided in two equal pieces and cultured separately in complete chondrocyte media for 48 hours after isolation. The weight of the explants ranged between 16 and 20 mg. One half was then subjected to 100 ng/mL Pam2CSK4 a TLR2/6 agonist (Invitrogen) in explant media (Dulbecco's DMEM with 4.5 g/L glucose (Sigma Aldrich), Insulin‐transferrin‐selenium (Invitrogen), 2 mmol/L Glutamax (Life Technologies), 25 µg/mL gentamycin (Life Technologies)) on a 20 volume (mL) per weight (g) of tissue (20× (v/w)) basis for 4 days while the other half served as untreated control in 20× (v/w) explant media. After the treatment period, media were collected, and samples taken for histology as described below.

### 2.4 Secreted protein analysis

Secreted S100A8/9 was measured in explant culture media using a Quantikine ELISA (R&D Systems). Secreted MMP3, MMP13, IL‐6 and IL‐8 were measured in explant culture media from tissue with or without TLR2 activation (Raybiotech). Measures were obtained using a VICTOR Nivo microplate reader (Perkin Elmer).

### 2.5 Histology and proteoglycan content analysis

Facet joint cartilage explants were fixed in 4% paraformaldehyde and cryoprotected in 10, 20 and 30% sucrose for OCT embedding and cryosectioning on a CryoStar NX70 cryostat (Thermo Fisher Scientific). 12 µm sections perpendicular to the joint were placed on SuperFrost plus slides (VWR). Slides were heated at 60°C for 30 minutes and rehydrated in PBS‐T (0.05% Tween 20 (Sigma Aldrich)) for 5 minutes. The slides were subsequently stained in 0.025% Safranin‐O (Sigma Aldrich) for 5 minutes, 1% glacial acetic acid for 15 seconds and finally in 0.01% fast green (Sigma Aldrich) for 5 minutes. Images of the sections were taken at 20× on a Leica DMRB microscope (Leica) with a DP70 digital camera (Olympus). The red safranin‐O staining was quantified using a MATlab script which normalizes background and measures the red pixel intensity in a delimited cartilage region of interest. Proteoglycan content released into the media was quantified using the Dimethylmethylene Blue (DMMB) assay.^34^

### 2.6 Mass spectrometry analysis

Conditioned media samples from the explant‐cultures were reduced with DTT and alkylated with iodoacetic acid. Following trypsinization and re‐solubilization in 0.1% aqueous formic acid and 2% acetonitrile, peptides were separated on a Thermo Acclaim Pepmap (Thermo, 75 µmol/L ID × 2 cm C18 3 µmol/L beads) precolumn followed by an Acclaim Pepmap Easyspray column (Thermo, 75 µmol/L × 15 cm with 2 µmol/L C18 beads) using a Dionex Ultimate 3000 uHPLC. Analysis of all peptides with a charge of 2+ or greater was detected with the Orbitrap Fusion mass spectrometer with HCD. The data were analysed and compared with known human proteins sequences (Swissprot) using Mascot 2.3. Results were compiled in Scaffold and illustrated using Graphpad prism.

### 2.7 In vitro* TLR antagonist evaluation*

Monolayer chondrocytes were used at passage 2. 150 000 cells were seeded in 6 well plates and were cultured in complete chondrocyte media to confluent. At confluence, cells were pre‐treated with 1/1000 dilution of Sparstolonin B, o‐Vanillin or PBS in serum‐free chondrocyte media for 1 hour before adding a final concentration of 1 μg/mL S100A8/9 (R&D Systems) or 2.5 µg/mL biglycan (R&D Systems). The synthetic agonist Pam2CSK4 (Invitrogen) was also used here as a positive control at a lower concentration of 1 ng/mL. The lower dose was chosen for physiological relevance to simulate the low‐grade inflammation found in OA. For gene expression analysis, cells were lysed in TRIzol 16 hours following alarmin treatment. Culture media were collected after 48 hours.

### 2.8 Statistical methods

For each comparison between scoliotic and non‐scoliotic groups or control and treated groups, a parametric Student's *t* test was used. For correlations between TLRs and degenerative factors, the Pearson method was used to assess the degree of relation and significance of *P* < .05. Each statistical method and correlation was calculated using GraphPad Prism.

## 3 RESULTS

### 3.1 Baseline gene expression of TLRs and degenerative factors

**Figure 1:**
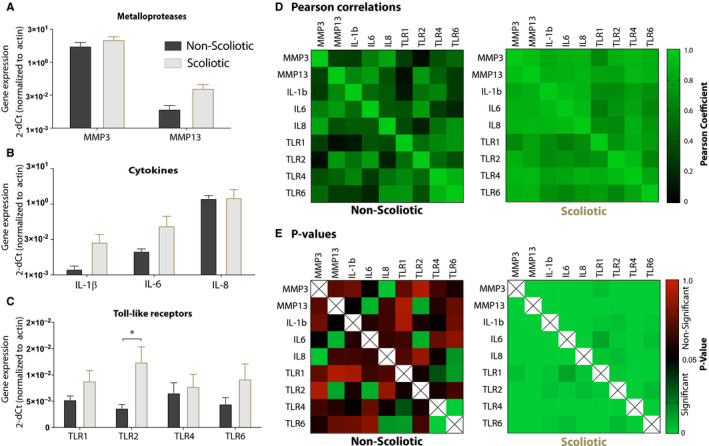
A‐C, Baseline MMP3, MMP13, IL‐1β, IL‐6 and IL‐8 and Toll‐like receptor 1,2,4 and 6 gene expression in scoliotic (n = 18) and non‐scoliotic (n = 12) facet joint chondrocytes. D, Pearson correlations of gene expression (cycle numbers) normalized to ß‐actin between TLR1, ‐2, ‐4, ‐6 and degenerative factors MMP3, MMP13, IL‐1β, IL‐6 and IL‐8. E, *P*‐values for each of the Pearson correlations in (D). Significance is evaluated at *P* < .05

The proteinases MMP3 and MMP13 and the cytokines IL‐1ß, IL6 and IL8 are often linked to OA in adults.^35^ We have demonstrated that young patients with AIS show evidence of facet joint OA but the expression of cytokines and proteases has not been well established. Here, we found the transcripts to be more abundant in scoliotic chondrocytes compared with non‐scoliotic (Figure 1A). MMP3 had a modest 1.28‐fold increase while MMP13 showed a 2.37‐fold higher expression in scoliotic chondrocytes. The pro‐inflammatory cytokine IL‐1ß had elevated mRNA levels in scoliotic chondrocytes, with a 2.97‐fold higher expression compared to non‐scoliotic samples (Figure 1B). A 1.98‐fold higher IL‐6 expression was seen scoliotic tissues and IL‐8 showed no difference between groups.

TLR 1, ‐2, ‐4, ‐6 are the main members involved in alarmin and danger signal recognition and the subsequent induction of pro‐catabolic and inflammatory mediators can contribute to tissue degeneration.^36^, ^37^ To evaluate the presence of this pathogenic mechanism in degenerating scoliotic cartilage, baseline mRNA expression of TLR receptors was evaluated from isolated chondrocytes (Figure 1C). Interestingly, all four TLRs evaluated were more abundant in scoliotic cartilage compared to non‐scoliotic, with TLR2 having the largest difference with a significant 2.84‐fold (*P* = .03) higher expression. In the scoliotic samples, TLR6 expression was 1.71‐fold higher, TLR1 expression was 1.49‐fold higher, and TLR4 expression was 1.15‐fold higher compared with non‐scoliotic samples. These data reinforce the knowledge of a catabolic and pro‐inflammatory state of degenerating facet joint chondrocytes with the added insight of an elevated TLR expression.

### 3.2 Gene expression correlation of TLRs and degenerative factors in scoliotic chondrocytes

A Pearson correlation analysis was performed to assess the relationship between TLR expression and factors linked to cartilage degeneration. The correlation analysis was performed between TLR1, ‐2, ‐4, ‐6, MMP3, ‐13, and IL‐1ß, ‐6, ‐8 gene expression levels, of scoliotic and non‐scoliotic samples separately (Figure 1D). Two parameters were used to evaluate the correlation of each gene pair: the Pearson coefficient details the correlation strength on a scale where 0 equates no correlation, .6 a moderate correlation and 1 being the strongest possible. A positive coefficient represents a proportional correlation whereas a negative coefficient an inversely proportional correlation. An overview of association results from scoliotic samples shows that all pairs of TLR (‐1,‐2,‐4,‐6) and degenerative factors (MMP3, MMP13, IL‐ß, IL‐6, IL‐8) correlated positively and significantly with each other (Figure 1D,E). Moreover, all correlations are strong (Pearson > .6) except the pair TLR1‐IL‐6 (Pearson = .547) which showed a moderate correlation. In stark contrast, gene expression in healthy non‐scoliotic cartilage was mostly independent from each other as only 8 out of 36 possible correlations were significant (Figure 1E). In order to better understand the relation in expression between individual TLRs, we also evaluated Pearson correlations between each TLR levels in scoliotic and non‐scoliotic chondrocytes. In scoliotic tissues, expression of the four TLRs was strongly (Pearson > .6) and significantly (*P* < .05) correlated. The strongest correlations were between TLR2‐TLR4 (Pearson = .899) followed by TLR4‐TLR1 (Pearson = .819) and TLR4‐TLR6 (Pearson = .780). For the non‐scoliotic samples, only 3 out of 6 pairs correlated significantly (TLR1‐TLR4, TLR1‐TLR6 and TLR4‐TLR6). Notably, TLR2 only had weak correlations with TLR1, ‐4 and ‐6, in non‐scoliotic cells. The strongest correlation in non‐scoliotic cells was between TLR4‐TLR6 (Pearson = .864, *P* = .0002) Together, the data indicate a strong relationship between TLR expression with inflammation and catabolism in degenerating facet joints.

### 3.3 TLR activation‐induced production of catabolic and pro‐inflammatory factors

**Figure 2:**
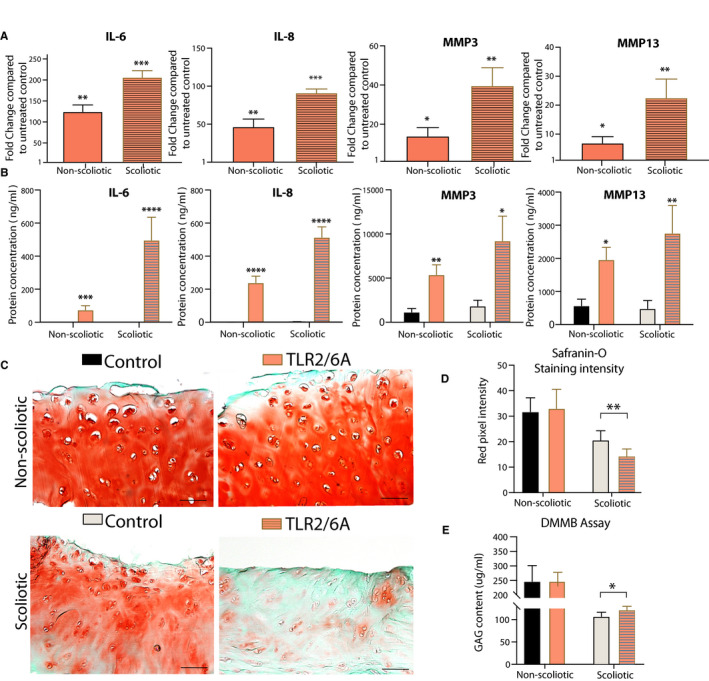
A, Gene expression analysis of TLR2/6 agonist (TLR2/6A) treated scoliotic and non‐scoliotic chondrocytes compare to non‐treated controls (n = 5). B, MMP3, MMP13, IL‐6 and IL‐8 secretion analysis of ex vivo facet joint cartilage cultured media from scoliotic (n = 10) and non‐scoliotic groups (n = 10) after 4 d of TLR2 activation with Pam2csk4. C, Safranin‐O fast green histology of scoliotic (n = 30) and non‐scoliotic (n = 13) facet joint cartilage before (Control) and after (Pam2csk4) TLR2 activation for 4 d. D, Red staining quantification for proteoglycan content using a MATlab script. E, GAG content analysis in the cultured media before and after Pam2CSK4 treatment. Paired Student *t* test were performed to assess significance defined by *P < .05, **P < .01, ****P < .001

To evaluate the effect of TLR activation, we subjected facet joint chondrocytes to the potent synthetic TLR2/6 agonist Pam2CSK4. Gene expression analysis revealed a significant increase of IL‐6, IL‐8, MMP3 and MMP13 in both scoliotic and non‐scoliotic chondrocytes (Figure 2A). Interestingly, scoliotic chondrocytes responded stronger than non‐scoliotic cells for all genes tested. IL‐6 was increased by 196‐fold (*P* = .0003) and IL‐8 by 91‐fold (*P* = .0001). The proteases MMP3 was increased 39‐fold (*P* = .0008) and MMP13 22‐fold (*P* = .0007) in scoliotic cells. Non‐scoliotic chondrocytes also responded significantly to the TLR2/6 agonist although to a lesser extent with a 117‐fold (*P* = .0009) and 46‐fold (*P* = .0008) increase in IL‐6 and IL‐8, respectively. MMP3 expression was increased by 13‐fold (*P* = .04) and MMP13 by sixfold (*P* = .03). To verify these finding in intact tissue, a cartilage explant experiment was conducted and protein secretion into culture media assessed by ELISA assays (Figure 2B). TLR2/6 activation significantly increased MMP3 secretion 5.7‐fold (*P* = .02) and 3.43‐fold (*P* = .006) from scoliotic and non‐scoliotic explants, respectively. For MMP13, the scoliotic samples produced more than the non‐scoliotic without activation. TLR2/6 activation significantly increased MMP13 expression with 5.39‐fold (*P* = .02) in scoliotic and by 4.63‐fold (*P* = .004) in non‐scoliotic explants. As IL‐6 production in untreated samples was under the detectable threshold of the assay, we could not determine a fold difference in production after TLR2/6 activation. However, both groups had detectable amounts after treatment demonstrating that IL‐6 production is also influenced by TLR2/6 activation. Finally, TLR2/6 activation significantly (*P* = 1.91 × 10^−6^) (*P* = 5.73 × 10^−5^)) increased IL‐8 protein expression in scoliotic and non‐scoliotic samples with 168‐fold and 289‐fold difference, respectively. Notably, scoliotic explants displayed the strongest induction of all 4 factors analysed after TLR2/6 activation. Scoliotic explants produced higher levels than non‐scoliotic, disregarding the initial amount measured in untreated controls.

### 3.4 TLR activation‐induced cartilage ECM degradation

Proteoglycan loss is a key feature of OA.^38^ To evaluate the effect of TLR2/6 activation on proteoglycan content, safranin‐O fast green staining was performed on ex vivo treated scoliotic and non‐scoliotic cartilage explants (Figure 2C). Proteoglycan content was measured semi‐quantitatively (Figure 2D). In scoliotic tissues, the cartilage had a lower baseline proteoglycan content compared with the non‐scoliotic cartilage and lost significantly more (*P* = .003) following TLR2/6 activation. In contrast, TLR2/6 activation did not cause an as robust proteoglycan loss in non‐scoliotic samples. Proteoglycan release in the culture media was measured with the DMMB assay and confirmed histological findings with increased release from scoliotic cartilage after TLR2/6 activation (Figure 2E). There was no significant difference in proteoglycan release in non‐scoliotic cartilage after TLR2/6 activation. Together, these findings support a role for TLR2/6 in the degenerative cycle of scoliotic facet joint cartilage.

### 3.5 Alarmins in scoliotic cartilage

**Figure 3:**
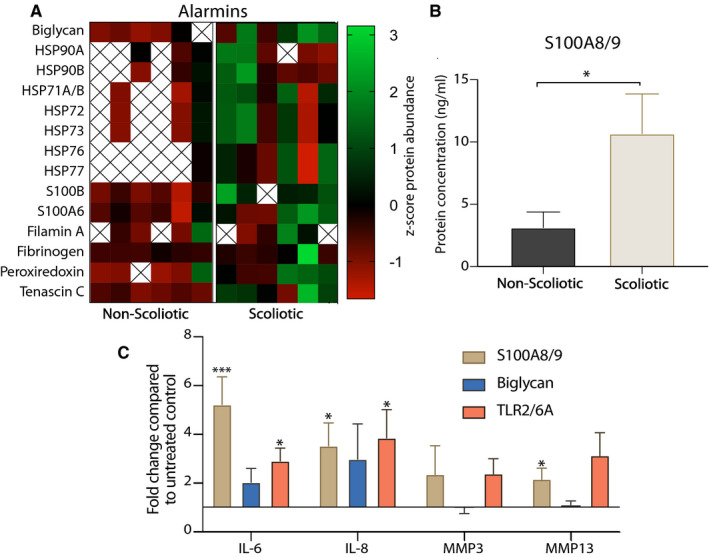
A and B, Mass spectrometry and ELISA of alarmin and S100A8/9 abundance in scoliotic and non‐scoliotic cartilage explant conditioned media (n = 6). C, Gene expression analysis of IL‐6, IL‐8, MMP3, MMP13 after stimulation by alarmins S100A8/9 and Biglycan and low‐dose TLR2/6 agonist treatment (TLR2/6A) in scoliotic facet joint chondrocytes (n = 4). Significance was evaluated by Student *t* tests (*P < .05, ***P < .001)

An increase in abundance of alarmins activating TLRs has been described in joints affected by OA in adults.^38^ Mass spectrometry and ELISA assays were used to reveal the alarmin secretion profile of scoliotic and non‐scoliotic cartilage (Figure 3A). The z‐score heat map shows an increased level of alarmins released from scoliotic cartilage. In contrast, protein levels were frequently below the detection threshold non‐scoliotic cartilage. Notably, cytoplasmic alarmins such as heat shock proteins (HSP) were found in all scoliotic samples but were mostly undetected in the non‐scoliotic group. S100A8/A9 has been strongly linked to adult OA.^39^ Although not detected with mass spectrometry analysis we set out to quantitively measure the concentration. Scoliotic cartilage released 2.9‐fold (*P* = .03) more S100A8/A9 than non‐scoliotic. The concentration was 10.33 ng/mL in scoliotic and 3.57 ng/mL in non‐scoliotic cartilage (Figure 3B). These results indicate an increased release of alarmins from degenerating scoliotic cartilage that could activate TLRs.

### 3.6 Alarmins‐induced pro‐inflammatory and catabolic factor production

Alarmins and TLR activation are believed to contribute to the chronic low‐grade inflammatory state present in adult OA.^40^ To evaluate the potential of naturally occurring alarmins to participate in facet joint cartilage degeneration, isolated scoliotic chondrocytes were subjected to S100A8/9 and biglycan, which are both released by scoliotic cartilage. A lowered concentration of the TLR2/6 agonist was used in these experiments to more accurately compare to the alarmin‐induced responses. As expected, the agonists caused an increase in IL‐6 an IL‐8 gene expression (Figure 3C). S100A8/A9 had the strongest effect on IL‐6 with a significant 5.14‐fold (*P* = .0009) increase. MMP3 and MMP13 gene expressions were only affected by S100A8/A9, with a 2.34‐fold increase. Biglycan treatment increased TLR2 expression with a modest 1.829‐fold increase. As expected, the low‐dose TLR2/6 agonist increased gene expression of the 4 genes to a similar level as found with S100A8/9. These results suggest a role for alarmins in scoliotic facet joint degeneration.

### 3.7 Sparstolonin B and o ‐Vanillin reduced alarmin‐induced TLR activation

**Figure 4:**
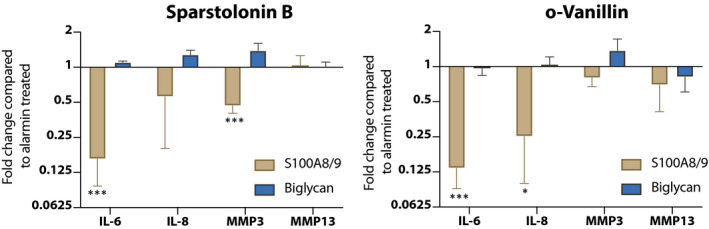
Gene expression analysis of degenerative factors under stimulation of alarmins S100A8/9 and biglycan in conjunction with antagonists Sparstolonin B and o‐Vanillin. Significance between control and treatment was evaluated at ***P < .001. Significance between alarmin and alarmin + antagonist was assessed at ±±± = *P* < .001 and ±± = *P* < .05

As TLR expression is elevated and the chondrocytes are strongly responsive to alarmins in scoliotic cartilage blocking their activity could potentially modify disease progression. To assess the effect of blocking TLR activation, two naturally derived compounds that prevent the recruitment of MyD88 to the TIR domain of TLRs, and thus TLR signalling, were used.^31^, ^41^ The antagonists did not significantly modulate gene expression in scoliotic chondrocytes challenged with biglycan (Figure 4A,B). However, Sparstolonin B and o‐Vanillin significantly suppressed S100A8/9‐induced IL‐6 gene expression by 6.12‐fold (*P* = .0009) and 7.43‐fold (*P* = .0007), respectively. IL‐8 expression decreased with both antagonists but significance was only reached with o‐Vanillin (3.98‐fold). In contrast, MMP3 expression was reduced by 2.14‐fold (*P* = .0008) with Sparstolonin B treatment, and MMP13 gene expression was unchanged by both antagonists, although a small decreasing trend after o‐vanillin treatment was found. This indicates that TLR inhibition overall reduces the alarmin‐induced pro‐inflammatory response.

### 3.8 Alarmins increased TLR gene expression

**Figure 5:**
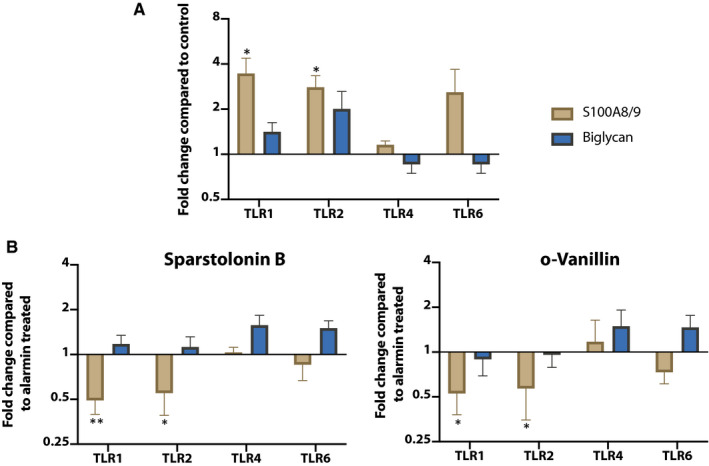
A, TLR gene expression after stimulation with alarmins S100A8/9 and Biglycan B) in conjunction with antagonists Sparstolonin B and o‐Vanillin. Significance between control and treatment was evaluated at *P < .05, **P < .01

TLR activation has been linked to an increased expression of TLRs. This mechanism can drive a vicious cycle in the presence of abundant alarmins such as in degenerating cartilage.^42^ We therefore evaluated TLR gene expression in response to activation in scoliotic cartilage. TLR4 gene expression was unchanged following biglycan, S100A8/9 and TLR2/6 agonist treatment. (Figure 5A). S100A8/9 had the strongest effect and induced TLR1, ‐2 and ‐6 expression by 6.242‐fold (*P* = .04), 2.799‐fold (*P* = .02) and 3.271‐fold, respectively, compared with untreated controls.

Treatment with Sparstolonin B and o‐Vanillin significantly reduced the levels of TLR1 and TLR2 expression induced by S100A8/9 (Figure 5B). TLR1 and TLR2 gene expression also decreased by 2.04‐fold (*P* = .007) and 1.81‐fold (p‐0.03) following Sparstolonin B treatment. TLR1 and TLR2 decreased with a similar magnitude, 1.9‐fold (*P* = .04) and 1.87‐fold (*P* = .03) following o‐Vanillin treatment. TLR4 and TLR6 expressions, however, were not affected by either of the antagonists. These results show the potential of using MyD88‐targeting small‐molecule inhibitors to block TLR signalling to potentially restore a homoeostatic balance in degenerating facet joints in AIS patients.

## 4 DISCUSSION

Our findings suggest that TLR activation is contributing to scoliotic facet joint degeneration. We found higher TLR gene expression and strong correlations with degenerative factors in scoliotic cartilage. TLR activation by synthetic agonists and naturally occurring alarmins induced the expression of pro‐inflammatory cytokines and proteases, which exacerbated cartilage breakdown in scoliotic facet joint cartilage. A potential beneficial effect of blocking TLR signalling was suggested from the effects of the MyD88 inhibitors Sparstolonin B and o‐Vanillin. Both reduced alarmin‐induced expression of proteases, pro‐inflammatory cytokines and TLR receptors.

A current hypothesis of the catabolic shift and chronic inflammation in osteoarthritic cartilage implicates the formation of alarmins through tissue degradation with a subsequent TLR activation.^43^ We confirmed that this mechanism is active in facet joints of young patients with scoliosis.

First, baseline gene expression for key catabolic factors and TLRs was measured. The increase in baseline gene expression of proteases MMP3 and MMP13 match extensive previous research showing a robust increase of these proteases in OA cartilage. Furthermore, it corroborates with immunohistochemistry findings in our previous study on scoliotic facet joint degeneration.^4^ Gene expression analysis of TLRs revealed that chondrocytes from degenerating scoliotic cartilage had an elevated baseline expression of TLRs compared with the non‐degenerate non‐scoliotic cells. These findings also align with other studies showing that TLR expression is up‐regulated in articular cartilage with increasing OA severity in adults.^25^ Notably, TLR2 had the largest and most significant difference between the two groups which is in accordance with data from osteoarthritic knee chondrocytes in adults.^26^ The amount of highly significant and strong correlations between TLRs (1,2,4,6) and degenerative factors (MMP3, MMP13, IL‐1ß, IL‐6, IL‐8) in scoliotic chondrocytes suggests that there is a strong link between TLR expression and degeneration in scoliotic cartilage. The fact that healthy non‐scoliotic cartilage lacked this strong correlation further supports this. We could only detect a significant correlation between TLR2 ‐MMP13 and TLR2‐IL‐6 in cells from non‐scoliotic tissue. One explanation could be that TLR2 homodimers are more prevalent in healthy tissues whereas TLR2/6 and TLR2/1 heterodimers are more prominent in scoliotic cartilage, but this would need to be confirmed. The fact that all TLRs were found to be up‐regulated in adult OA cartilage could further support why they correlate strongly with each other only in the scoliotic samples.^25^

The responsivity of facet joint chondrocytes to TLR2/6 activation was strong, as evidenced by large fold‐changes when compared to untreated controls. As expected, cytokines were the most increased after activation in both groups. The stronger response in cells from scoliotic tissue might be a reflection of elevated expression levels of TLRs detected. The ex vivo cartilage explant experiment supports the gene expression analysis of isolated cells confirming elevated levels of protease and cytokine release into the media after TLR2/6 activation. The robust induction of proteases and cytokines in cells from scoliotic samples following TLR activation indicates that they are more susceptible to TLR‐induced joint catabolism. Ex vivo cartilage explants subjected to TLR2/6 activation revealed a pronounced proteoglycan loss in scoliotic samples, which supports the susceptibility to TLR activation in scoliotic cartilage. Gene expression analysis of aggrecan suggests that the proteoglycan loss is not due to a change in synthesis as gene expression levels did not change after TLR activation (Data not shown). The induced expression of proteases (MMP3, MMP13) and pro‐inflammatory cytokines (IL‐6, IL‐8) likely contributed to this drastic catabolism. Proteoglycan loss from the tissue and release to culture media was not affected in non‐scoliotic explants and might reflect on three possibilities. First, the semi‐quantitative measurement of proteoglycan staining might not be sensitive enough to detect the loss from healthy tissue with a much higher initial content than what is present in scoliotic cartilage. However, there was also no change proteoglycan released to the culture media. Secondly, as non‐scoliotic cells have a lower baseline expression of TLRs, the short exposure of 4 days to the agonist might not have been enough to allow for receptor up‐regulation and subsequent release of degenerative factors. Thirdly, the agonist might not readily penetrate healthy cartilage to induce degenerative factors. However, isolated non‐scoliotic cells also responded less to the agonist. Other studies have shown proteoglycan loss following TLR activation in non‐degenerate cartilage and a longer treatment period would likely have resulted in the loss seen with scoliotic samples. The literature also supports a role for MMP13 in OA, where overexpression of MMP13 was shown to be sufficient to cause osteoarthritis in mice.^44^ Another plausible explanation is a modulation of TLR signalling by a yet unknown mechanism in scoliotic facet joint OA, which should be investigated further.

To better assess the potential of TLR activation in degenerating scoliotic facet joints, we quantified the alarmin profile. The observable trend of increased abundance of alarmins in scoliotic samples is corroborated by studies showing increased alarmins in degenerating cartilage of adults.^38^ The alarmins detected has been described in the literature to activate TLR1,‐2,‐4, or ‐6. For example, the extracellular matrix molecule biglycan interact with TLR2 and TLR4 and induces pro‐inflammatory molecules in macrophages.^20^ S100A8/9, which was released at a significantly higher level from scoliotic cartilage, has been described to induce pro‐inflammatory responses in macrophages.^19^ Here, we evaluated the effect of biglycan and S100A8/9 in scoliotic chondrocytes where treatment increased gene expression of IL‐6, IL‐8, MMP3 and MMP13, which is similar to experiments done OA chondrocytes.^39^ The alarmins had a similar response to a low concentration of the synthetic TLR2/6 agonist, mimicking a chronic low‐grade inflammation found in OA. The difference in gene expression induction between high and low concentration of the synthetic TLR2/6 agonist reflect an increase proportional to concentration. Further studies are needed to fully elucidate the detrimental role of TLR activation following exposure to alarmins, as every alarmin has a different binding potential that could result in varying downstream effects.

Although this study suggests the involvement of TLRs in scoliotic facet joint OA, the clinical relevance of blocking TLRs to slow disease progression still needs to be evaluated. In support of our suggestion, a joint‐saving outcome of blocking TLRs has been described in an experimental osteoarthritis models.^45^ The successful reduction of alarmin‐induced protease and pro‐inflammatory cytokine production by Sparstolonin B and o‐Vanillin opens the possibility of using small‐molecule inhibitors to suppress chronic inflammation and catabolism in scoliotic facet joints. Therefore, blocking TLR signalling should be studied further to explore a potential disease‐modifying effect of such inhibitors.

A limitation in this study was the age differences between scoliotic and non‐scoliotic subjects. The scoliotic cohort had a lower and homogeneous age range whereas non‐scoliotic organ donors had a wider age range and a higher average age. This is explained by the window at which patients with AIS undergo corrective surgery and the variable age of organ donors. However, there was a variability in curve severity and degree of OA in AIS patients whereas all non‐scoliotic organ donors had healthy spines with no signs of OA and no prior spine deformities. Therefore, we believe that the heterogeneity in age is counterbalanced by the clear differences in cartilage health between the groups.

## 5 CONCLUSIONS

In conclusion, our results suggest that TLRs are integral to cartilage degeneration in scoliotic facet joints. The higher baseline expression of TLRs in scoliotic cartilage and the strong and significant correlation with proteases and pro‐inflammatory cytokines suggests that they are key regulators of tissue degradation. Taken together, these findings provide an insight into a potential target for future molecular therapies aiming at restoring tissue homoeostasis and prevent tissue degradation and loss of function.

## CONFLICTS OF INTEREST

The authors have no conflict of interest to disclose. Dr Lisbet Haglund and Dr Jean Ouellet were jointly awarded a grant from the Shriners Hospitals for Children (Montreal, Canada) to perform the studies.

## AUTHOR CONTRIBUTION

**Daniel G. Bisson:** Conceptualization (equal); Data curation (equal); Formal analysis (equal); Investigation (equal); Methodology (equal); Writing‐original draft (equal); Writing‐review & editing (equal). **Kai Sheng:** Data curation (equal); Formal analysis (equal); Methodology (equal). **Semsi Kocabas:** Data curation; Formal analysis. **Emerson Krock:** Data curation (equal); Formal analysis (equal). **Alisson Teles:** Resources (equal). **Neil Saran:** Resources (equal); Writing‐review & editing (equal). **Jean A. Ouellet:** Conceptualization (equal); Funding acquisition (equal); Investigation (equal); Project administration (equal); Resources (equal); Supervision (equal); Validation (equal); Writing‐review & editing (equal). **Lisbet Haglund:** Conceptualization (equal); Funding acquisition (equal); Investigation (equal); Methodology (equal); Project administration (equal); Resources (equal); Supervision (equal); Writing‐original draft (equal); Writing‐review & editing (equal). 

## Data Availability

The data generated in this study are available from the corresponding author on reasonable request.
